# Response of Human Neutrophil Granulocytes to the Hyphae of the Emerging Fungal Pathogen *Curvularia lunata*

**DOI:** 10.3390/pathogens9030235

**Published:** 2020-03-21

**Authors:** Eszter Judit Tóth, Mónika Varga, Miklós Takó, Mónika Homa, Olivér Jáger, Edit Hermesz, Hajnalka Orvos, Gábor Nagy, Csaba Vágvölgyi, Tamás Papp

**Affiliations:** 1 MTA-SZTE Fungal Pathogenicity Mechanisms Research Group, Hungarian Academy of Sciences—University of Szeged, 6726 Szeged, Hungary; forimistio@gmail.com (E.J.T.); homamoni@gmail.com (M.H.); jager.oliver.biology@gmail.com (O.J.); nagygab86@gmail.com (G.N.); 2 Department of Microbiology, Faculty of Science and Informatics, University of Szeged, 6726 Szeged, Hungary; varga.j.monika@gmail.com (M.V.); tako78@bio.u-szeged.hu (M.T.); csaba@bio.u-szeged.hu (C.V.); 3 Department of Biochemistry and Molecular Biology, Faculty of Science and Informatics, University of Szeged, 6726 Szeged, Hungary; hermeszedit@gmail.com; 4 Department of Obstetrics and Gynecology, Faculty of Medicine, University of Szeged, 6725 Szeged, Hungary; orvosh@obgyn.szote.u-szeged.hu

**Keywords:** *Curvularia*, phaeohyphomycosis, neutrophil extracellular trap, oxidative burst, myeloperoxidase, hydrogen peroxide, acidification

## Abstract

*Curvularia lunata* is an ascomycete filamentous fungus causing local and invasive phaeohyphomycoses in both immunocompromised and immunocompetent patients. Neutrophils are crucial participants of the first line host defense against fungal infections. They migrate to the infected site and eliminate the infectious agents by various mechanisms including phagocytoses, oxidative damage, or formation of neutrophil extracellular trap (NET). Neutropenia may be a risk factor for phaeohyphomycoses, and restoration of the neutrophil function can improve the outcome of the infection. In the present study, interaction of primary human neutrophil granulocytes with the hyphae *C. lunata* was examined and compared to that with the well characterized filamentous fungal pathogen *Aspergillus fumigatus*. Neutrophils could recognize the serum opsonized hyphae of *C. lunata* and attach to them. Myeloperoxidase release was also activated by a soluble factor present in the culture supernatant of the fungus. Induction of the oxidative burst was found to depend on serum opsonization of the hyphae. Although extracellular hydrogen peroxide production was induced, the fungus efficiently blocked the oxidative burst by acidifying the reaction environment. This blockage also affected the NET forming ability of the neutrophils.

## 1. Introduction

*Curvularia* is an ascomycete fungal genus, which mainly includes saprotrophic and plant pathogenic species. However, it is also considered as one of the most relevant filamentous fungal group involved in local and invasive phaeohyphomycoses, i.e., human infections caused by dematiaceous fungi [1,2,3,4]. *Curvularia* infections can manifest as mycotic keratitis [5], cutaneous and subcutaneous mycosis [6], onychomycosis [7], peritonitis [8], and allergic sinusitis [8,9]. Deep infections frequently affect the central nervous system [8,9,10]. Development of disseminated mycoses has been reported in both immunocompromised and immunocompetent patients [11,12,13,14]. Within the genus, *C. lunata* is among the species most frequently identified from clinical samples [5,15]. Despite their abovementioned role as the causative agents of phaeohyphomycoses, there are only very limited information available about the immunological aspects and the pathomechanisms of *Curvularia* infections [16].

Immunosuppression and immunodeficiencies result in predisposition to opportunistic fungal infections [17], including those of *Curvularia* species [4]. Action of monocytes and macrophages phagocyting the conidia and neutrophils causing hyphal damage has a key role in the prevention of invasive mycotic infections [18,19]. Neutrophils are among the first immune cells, which migrate to the infected site and they eliminate the microbes by various mechanisms involving phagocytoses, formation of neutrophil extracellular trap (NET) or release of oxidative molecules (such as hydrogen peroxide or hydroxyl radicals), enzymes or antimicrobial peptides [20,21]. Action of neutrophil granulocytes against *Aspergillus* is a well-studied field of host–fungal pathogen interactions [17,22,23] but little is known about their role in the defense to *Curvularia* species and other dematiaceous molds. Neutropenia was found to be associated with high risk of death in phaeohyphomycosis, while recovery from neutropenia improved the chance of the survival [24].

In a recent study, we examined the interaction of *C. lunata* with human monocytes and compared it to that of *A. fumigatus* and other *Curvularia* species [16]. In that study, monocytes attached to the hyphae of *C. lunata* produced interleukin (IL)-8 in an elevated level. The primary role of this cytokine is to recruit neutrophils to the site of infection [25]. Here, we report the interaction of *C. lunata* with human neutrophil granulocytes, which represent another key component of the first line defense in the elimination of the fungal pathogens.

## 2. Results

### 2.1. Activation of the Neutrophils by the C. lunata Hyphae

**Figure 1:**
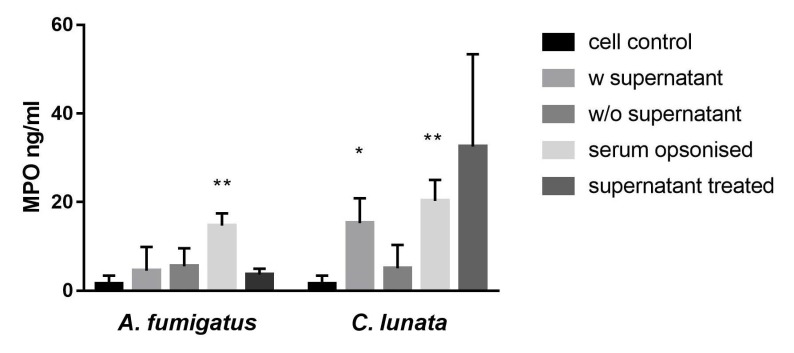
Neutrophil granulocytes are activated by serum opsonized *C. lunata* and by the culture supernatant of the hyphae. Myeloperoxidase (MPO) release of neutrophil granulocytes was measured after 60 min of interaction with *A. fumigatus* and *C. lunata*. Cell control: untreated neutrophils; w supernatant: neutrophils were added directly to the germinated conidia; w/o supernatant: germinated conidia were washed with fresh mKRPG before addition of neutrophils; serum opsonized: young hyphae were serum opsonized before addition of neutrophils; supernatant treated: cell free culture supernatant of the germinated conidia was added to the neutrophils. Error bars represent standard deviations calculated from three biological parallels. Significance was calculated with multiple t-test compared to the cell control using the GraphPad Prism 7 software, FDR (Q = 10%), * *p* < 0.05 ** *p* < 0.0001.

The culture supernatant of *C. lunata* with the hyphae and the serum opsonized hyphae induced the myeloperoxidase (MPO) release of the neutrophils (Figure 1). If the culture supernatant was changed to modified Krebs-Ringer phosphate glucose (mKRPG) buffer over the hyphae, MPO release did not increase compared to the untreated neutrophils. Presence of the *A. fumigatus* hyphae induced significant MPO release only after serum opsonization, while the culture supernatant of the germinated conidia could not activate the neutrophils.

### 2.2. Generation of Hydrogen Peroxide (H_2_O_2_)

**Figure 2:**
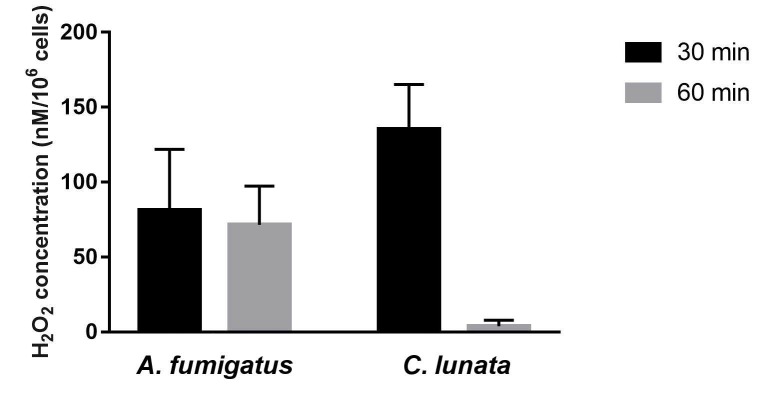
The amount of H_2_O_2_ decreases after 60 min of interaction with *C. lunata*. H_2_O_2_ concentration was measured after 30 and 60 min of interaction with the serum opsonized hyphae of *A. fumigatus* and *C. lunata* and it was normalized to 10^6^ neutrophils. Error bars represent standard deviations calculated from three biological parallels.

Both *C. lunata* and *A. fumigatus* hyphae induced the H_2_O_2_ release only after opsonization (Figure 2). Interestingly, the released H_2_O_2_ almost completely disappeared at the 60th min of the interaction with *C. lunata*, while its level proved to be more or less constant in the case of *A. fumigatus*. Without the fungi, H_2_O_2_ release of the neutrophil cell could not be detected.

### 2.3. Detection of Extracellular Melanin

As *C. lunata* hyphae can produce dihydroxynaphthalene (DHN)-type melanin, which may affect the oxidative damage by the neutrophils, melanin production during the interaction was tested. Acidification causes precipitation of the melanin pigment. However, by acidifying the supernatant of the interaction, no precipitation was formed, and no visible residue left on the polytetrafluoroethylene (PTFE) membrane. Acidification and filtering of the culture media from 7 days long cultivations in mKRPG showed the same result.

### 2.4. NET Formation in the Presence of the Hyphae

**Figure 3:**
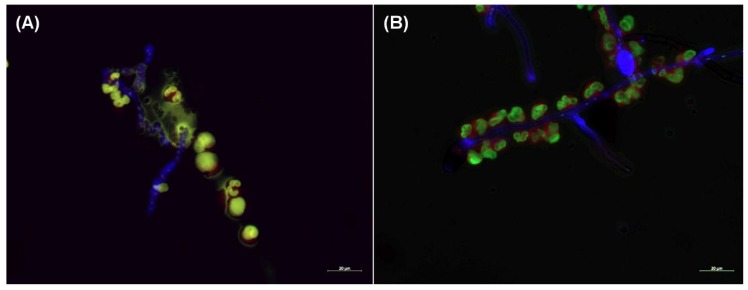
Neutrophil extracellular trap (NET) formation is missing when neutrophils are confronted with the hyphae of *C. lunata*. Fluorescent microscopy was performed about after 3 h of interaction of serum opsonized *A. fumigatus* (**A**) and *C. lunata* (**B**) with neutrophil granulocytes. Hyphae and conidia were labeled with Calcofluor white (blue); neutrophil’s cell membrane was stained with CellMask Deep Red Plasma Membrane Stain (red) and the DNA was stained with SYTOX Green (green). NET formation was detected in the interaction with *A. fumigatus* but only gathering of the cells to the hyphae could be observed in case of *C. lunata*.

Serum opsonized *A. fumigatus* hyphae induced the NET formation, as it was published previously [17] (Figure 3a). In contrasts, NET formation was not observed in the presence of the *C. lunata* hyphae (Figure 3b).

**Figure 4:**
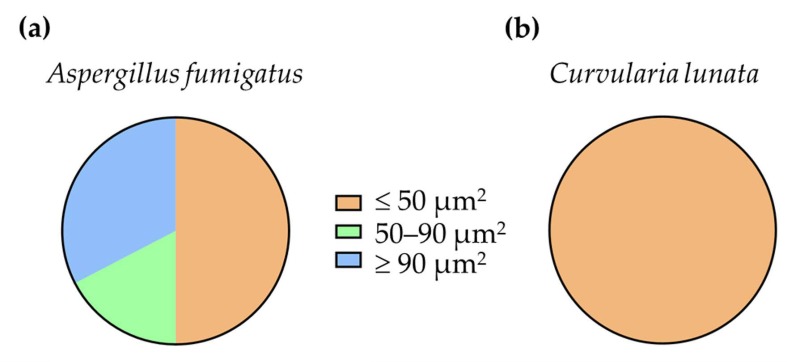
Ratio of the nuclear areas measured after 3 h of interaction of serum opsonized *A. fumigatus* (**a**) and *C. lunata* (**b**) with neutrophil granulocytes. Nuclear areas of ≤50 μm^2^ were considered as condensed nuclei, those which were between 50 and 90 μm^2^, were regarded as decondensed nuclei, while those measured to be ≥90 μm^2^ indicated NET formations.

It was even not induced if H_2_O_2_ was added to the interactions. Seemingly, neutrophils recognized the serum opsonized hyphae and attached to it, but the lobular structure of the nuclei remained intact and no chromosome decondensation was observed. After 3 h of co-incubation with the neutrophils, the 17% and 33% of the nuclear areas of *A. fumigatus* indicated decondensation and NET formation, respectively (Figure 4). At the same time, 100% of the nuclei remained condensed in case of *C. lunata*. Without serum opsonization, neutrophils did not gather around the hyphae of both fungi.

### 2.5. Extracellular pH of the Neutrophil–Fungus Interaction

**Figure 5:**
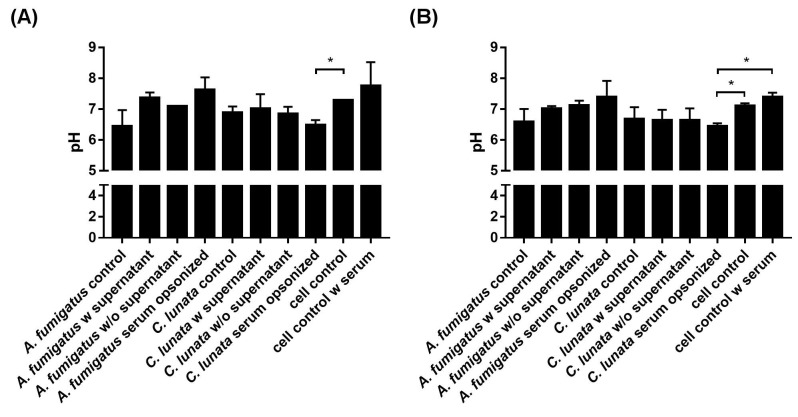
Extracellular pH of the environment after 60 min (**A**) and 3 h (**B**) of interaction with *A. fumigatus* and *C. lunata*. Addition of serum alkalifies the media; significance of serum opsonization conditions were calculated compared to the untreated neutrophils with and without serum as well. Control: untreated hyphae or neutrophils; w supernatant: neutrophils were added directly to the germinated conidia; w/o supernatant: germinated conidia were washed with fresh modified Krebs-Ringer phosphate glucose (mKRPG) buffer before addition of neutrophils; serum opsonized: young hyphae were serum opsonized before addition of neutrophils. Error bars represent standard deviations calculated form three biological parallels. Multiple t-test was used in GraphPad Prism 7 software, FDR (Q = 10%), * *p* < 0.05.

Infection of the neutrophils with *A. fumigatus* did not change significantly the extracellular pH compared to that of the uninfected cells at the tested timepoints (Figure 5a). However, serum opsonized hyphae of *C. lunata* decreased the pH of the interaction environment even after 60 min and this decrease proved to be significant after 3 h of interaction (Figure 5b).

### 2.6. Killing Efficiency of the Neutrophil Granulocytes

**Figure 6:**
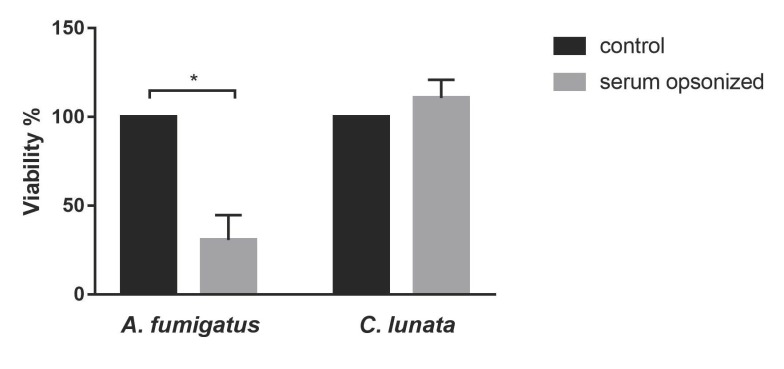
Viability of *C. lunata* is not affected by 3 h-long interaction with neutrophils. Neutrophils were added to the serum opsonized hyphae. As a control, fungal strains were cultivated under the same conditions without immune cells. Viability is expressed as the percentage normalized to the control conditions. Error bars represent standard deviations calculated from three biological parallels. Significance was calculated with multiple t-test with False Discovery Rate (FDR) (Q = 10%) in the GraphPad Prism 7 software, * *p* < 0.05.

While the neutrophils could effectively kill *A. fumigatus* in case of serum opsonization, viability of *C. lunata* did not decreased after 3 h of interaction under the examined conditions (Figure 6).

### 2.7. Viability of the Neutrophils in the Presence of the Fungal Hyphae

At 60 min post-inoculation, significant death of the neutrophils was detected only in the presence of serum opsonized hyphae. *C. lunata* and *A. fumigatus* caused a cell death of 68% and 26% compared to the control, respectively.

## 3. Discussion

Neutrophils are key components of the host defense against fungal pathogens and neutropenia or inadequate neutrophilic response generally are considered as risk factors of opportunistic fungal infections. Therefore, the interaction of neutrophil granulocytes with opportunistic human pathogenic fungi is an intensively researched field [26]. In this respect, the most studied filamentous fungus is *A. fumigatus* [27]. Patients having chronic granulomatous disease (CGD), which is caused by a deficiency in the NADPH-oxidase system of phagocytes, making them unable to produce reactive oxygen species, are at high risk having invasive aspergillosis (IA) or other fungal infection. CGD also affects the NET forming capacity of neutrophils [17,28]. Disseminated phaeohyphomycoses including those caused by *Curvularia* spp. also occur with a higher prevalence in immunosuppressed patients, in which case, the survival rate was found to be higher when the patient recovered from neutropenia (24).

It is known that neutrophils recognize the *A. fumigatus* hyphae only after serum opsonization, and efficient killing mechanisms are then activated [17]. Consistently to this, activation of the neutrophils in the presence of the *A. fumigatus* hyphae could be observed only after opsonization. Opsonization of the *C. lunata* hyphae also led to the MPO release of the neutrophils indicating that the recognition may be similar to that of *A. fumigatus.* The fact that we could detect release of MPO when the supernatant of the hyphae was not removed before the infection or even only the supernatant was added to the neutrophil cells suggests that neutrophils could also recognize a soluble factor produced by *C. lunata*.

MPO forms a complex with the H_2_O_2_ produced during respiratory burst, which is responsible for the oxidation of halides forming hypohalous acids like hypochlorous acid (HOCl) [29,30]. NADPH oxidase of neutrophils produce high amount of superoxide (O_2_^-^), which is converted into H_2_O_2_ by superoxide dismutase or by a spontaneous reaction [20]. By measuring the amount of H_2_O_2_ produced by the neutrophils in response to fungi, we observed that oxidative burst was activated only in the presence of the opsonized hyphae of both fungi. However, while the H_2_O_2_ level was quite constant in case of *A. fumigatus*, it reduced radically in the presence of *C. lunata*, indicating a potent antioxidant activity for the latter fungus.

*C. lunata* produces DHN-type melanin. Antioxidant capacity of melanin is well documented [31]. Moreover, the protective effect of melanin was proven against H_2_O_2_ and HOCl in the case of *A. nidulans* [32]. However, as the presence of released melanin was not detected in the interaction environment, it can be suggested that the drop of the H_2_O_2_ level was not the consequence of the antioxidant activity of this pigment in our study.

Large microbes and fungal hyphae trigger extracellular reactive oxygen species (ROS) release, which leads to recruiting further neutrophils and NET formation [33]. Interestingly, we detected H_2_O_2_ production in not all those cases when activation of neutrophils was seen. MPO release induced by the supernatant of the hyphae did not result in H_2_O_2_ production. MPO has important roles in the proinflammatory regulation. In a non-enzymatic way, it activates further neutrophils via an autocrine signaling pathway [34], mediates the recruitment of other leukocytes binding to their CD11b/CD18 receptors [35], and induces the IL-6 and IL-8 production of endothelial cells [36]. It is possible that the MPO functions as a regulating molecule when cells are activated by the soluble factor of *C. lunata*.

NET formation was observed in the presence of opsonized *A. fumigatus* but not in case of *C. lunata*, although neutrophils attached to the hyphae. NET formation is mainly regulated by the presence of ROS [37]. It is not clear if there is a specific ROS regulating the process. According to Nishinaka [38], singlet oxygen is the main inducer of NET production, but there are also data contradicting this hypothesis [39,40]. In parallel with the lack of NET formation, an acidification of the reaction environment was observed in the case of *C. lunata*. It was recently reported that acidification of the environment can inhibit the NET formation of neutrophils via the impeding of ROS production, but addition of H_2_O_2_ was enough to induce NET appearance [41]. Considering that H_2_O_2_ almost disappeared and the extracellular pH was found around 6.5 during the interaction with *C. lunata*, this can explain the lack of NET formation in response to this strain.

Viability of fungal strains was determined after three hours of interaction. While neutrophils were able to kill *A. fumigatus* efficiently, viability of *C. lunata* did not decrease after the interaction. This data is in line with our other results, suggesting that *C. lunata* can efficiently block the neutrophil functions. Cell death of neutrophils was also measured after the interaction. In the presence of *C. lunata*, neutrophils were more viable than those in the interaction with *A. fumigatus*. This result is in agreement with the observation that acidification of the reaction environment prolongs the lifespan of neutrophils [41] and supports the possible role of acidification in the protection of the fungus against neutrophil effector functions.

In a previous study, we found that THP-1 monocytes responded only to the hyphae of *C. lunata* by attaching to them and producing elevated levels of the anti-inflammatory cytokine IL-10 [16]. Induction of IL-10 together with the observation that monocytes could not reduce the viability of the fungus correlates well to the fact that *C. lunata* frequently cause chronic infections even in immunocompetent persons [8]. Neutrophils also could not decrease the fungal viability and their NET formation was blocked by the hyphae. This efficient evasion of the monocytic and the neutrophil attacks may contribute in the ability of this fungus to cause disseminated infections even in immunocompetent patients [8,11,12,14].

## 4. Materials and Methods

### 4.1. Fungal Strains, Culture Conditions, and Inoculum Preparation

The fungal strains, *Curvularia lunata* SZMC 23759 and *Aspergillus fumigatus* SZMC 23245 isolated from human eye infections were used (SZMC, Szeged Microbiology Collection, Szeged, Hungary; http://www.wfcc.info/ccinfo/collection/by_id/987). To harvest the conidia, fungi were grown on rice flour agar (1% rice flour, 0.2% yeast extract, 1.5% agar) at room temperature. Conidia were washed from 7 days old colonies with modified Krebs-Ringer phosphate glucose (mKRPG) buffer (145 mM NaCl, 5.7 mM Na_2_HPO_4_, 4.86 mM KCl, 5.5 mM glucose, pH 7.35) and the spore suspensions were filtered through a Millipore filter with a pore size of 45 μm (Merck, Burlington, USA) to eliminate the hyphal debris.

### 4.2. Isolation of Human Neutrophil Granulocytes and Serum

Human neutrophils were isolated from heparinized venous blood of healthy donors (n = 6) with dextran (Sigma-Aldrich, St. Louis, MI, USA) sedimentation followed by centrifugation over Ficoll-Paque PLUS (GE Healthcare, Chicago, IL, USA) and hypotonic lysis of erythrocytes [42]. Isolation was performed at room temperature. After isolation, the cells were resuspended in mKRPG. Viability and purity of the cells were checked with trypan blue (Sigma-Aldrich, St. Louis, MI, USA) [43] and Wright staining (Sigma-Aldrich, St. Louis, MI, USA) [44,45], respectively. Serum was isolated from venous blood of the same donors taken into serum separation blood collection tubes (BD Vacutainer, Becton Dickinson, Franklin Lakes, NJ, USA). Then, coagulation tubes were centrifuged at 1200 rpm for 15 min at room temperature and the serum was collected.

### 4.3. Interaction of the Neutrophils and the Hyphae

To produce young hyphae, 2 × 10^4^ conidia were inoculated in 100 µL mKRPG in a 96-well plate (Sarstedt, Nümbrecht, Germany) and incubated at 37 °C. Considering that the time of the germ tube formation from the conidia is different for *Aspergillus* and *Curvularia*, the incubation time was set to 4 and 8 h in case of *C. lunata* and *A. fumigatus*, respectively, to obtain the same length of hyphae for both strains. The isolated neutrophils were added to the germinated conidia in an effector:target (E:T) ratio of 5:1 in 100 µL mKRPG in a 96-well plate. Interactions were examined after 30, 60, or 180 min under three conditions: (a) neutrophils were added directly to the germinated conidia; (b) neutrophils were added to germinated conidia previously washed with fresh mKRPG buffer; and (c) neutrophils were added to the germinated conidia, which was previously serum opsonized with 10% serum of the corresponding donor in the last hour of germination. For detection of neutrophil activation, a fourth condition was also examined, in which the cell free supernatant of the germinated conidia was added to the neutrophils. Final volume of the interactions was 200 µL.

To detect possible NET formation, 7 × 10^4^ conidia were germinated in CELLview dishes (Greiner Bio-One, Mosonmagyaróvár, Hungary) in 400 µL mKRPG buffer and neutrophils were added to them in an E:T ratio of 5:1. Final volume of the reaction was 800 µL.

### 4.4. Detection of Neutrophil Activation

To measure the activation of neutrophils, the MPO activity was determined using 3,3′,5,5′-tetramethylbenzidine (TMB; Sigma-Aldrich, St. Louis, MI, USA) at the 30th and 60th min of the co-incubation according to the method of Suzuki et al. [46]. To exclude other peroxidase activities from the measurement, the MPO inhibitor 4-aminobenzoic hydrazide (ABH) was added to a parallel measurement in a concentration of 1 mM. MPO activity of the untreated neutrophils was considered as a control. The standard curve was generated based on the reaction of Recombinant Human Myeloperoxidase Protein (R&D Systems, Minneapolis, MN, USA) using 4-parametric logistic regression.

### 4.5. Measurement of the H_2_O_2_ Release of Neutrophils

Release of H_2_O_2_ was measured with the Amplex Red Hydrogen Peroxide/Peroxidase Assay Kit (Invitrogen, Carlsbad, CA, USA) 30 and 60 min after the infection according to the manufacturer’s recommendation.

### 4.6. Detection of Extracellular Melanin Release

Extracellular melanin release of *C. lunata* was analyzed during the fungi–neutrophil granulocyte interaction, as well as after the cultivation of the fungi in mKRPG for 7 days. Conditions of the interaction were the same as described in the Section 4.3. The supernatant from the interaction was centrifuged at 6000 rpm for 10 min, while the mKRPG from the cultivation was filtered through gauze. The flowthrough was acidified to pH 2.0 with addition of HCl and the resulting precipitate was filtered on a 0.2 µm PTFE membrane [47].

### 4.7. Visualization of NET Formation

NET formation was analyzed after 3 h of co-incubation (the E:T ratio was 5:1). Samples were fixed with 1% formaldehyde solution for 15 min.

To measure the chromosome decondensation and visualize the netosis, staining solutions were prepared in 10 mM sodium citrate buffer (pH 7.2). To stain the NET, 167 nM SYTOX Green nucleic acid stain (Life Technologies, Carlsbad, CA, USA) was applied for 30 min, while the fungal and the neutrophil cells were stained with 5 µg/mL Calcofluor white (Cyanamid, NJ, USA) for 15 min and a 0.5-fold solution of the CellMask Deep Red Plasma Membrane Stain (Thermo Scientific, Waltham, MA, USA) for 15 min, respectively. Between the staining steps and after the whole process, samples were washed two times with 10 mM sodium citrate buffer.

To induce NET formation by hydrogen peroxide (H_2_O_2_), the latter was added to the interactions after 0, 1, and 2 h of co-incubation in a final concentration of 30 μM [41].

Microscopic examinations were carried out with an Axio Observer 7 (Zeiss, Oberkochen, Germany) fluorescent inverted microscope.

### 4.8. Measurement of Chromosome Decondensation

Chromosome decondensation was analyzed on the pictures of samples stained with SYTOX green, Calcofluor white, and CellMask Deep Red and taken by fluorescent microscopy. For each sample, at least 35 cells were analyzed. SYTOX Green positive area was measured with ImageJ for each cell as described before [48]. Nuclear areas found to be ≤50 μm^2^ were considered as condensed nuclei, those, which were between 50 and 90 μm^2^, were regarded as decondensed nuclei, while those measured to be ≥90 μm^2^ indicated NET formations.

### 4.9. Measurement of Extracellular pH

Extracellular pH was determined from the cell-free supernatant of the interaction at 60 min and 3 h post-inoculation with litmus solution (CPAChem, Stara Zagora, Bulgaria). Calibration curve was generated from a pH scale of mKRPG with the linear regression model. To 100 µL of supernatant or mKRPG 5 µL of 2% litmus solution was added in a 96-well plate. Absorption was measured at 580 nm with a SPECTROstar Nano Microplate Reader (BMG Labtech, Ortenberg, Germany).

### 4.10. Viability Assay for the Hyphae

To measure the hyphal damage after incubation with the neutrophils, a colorimetric assay using 3-(4,5-dimethylthiazol-2-yl)-2,5-diphenyltetrazolium bromide (MTT; Sigma-Aldrich, St. Louis, MI, USA) was performed in 96-well plates [49]. After 3 h of coincubation, neutrophils were lysed with 0.5% sodium deoxycholate (Sigma-Aldrich) and the wells were washed three times with PBS. Two hundred µL of mKRPG buffer supplemented with 0.5% MTT was added to the samples and the plates were incubated at 37 °C for 3 h. After removing the supernatant, wells were washed two times with PBS and stored overnight at −20 °C. Before detection, 200 µL acidic isopropanol (95% isopropanol and 5% 1 N HCl) was added into the wells and the plates were incubated until the blue color dissolved from the hyphae. Absorbance of the supernatant was measured at the wavelength of 550 nm using a spectrophotometer. Viability of the hyphae was calculated using the following formula, where *OD*_550_ *sample* was the absorbance measured for the hyphae incubated with neutrophils and *OD*_550_ *control* was the absorbance measured for the hyphae incubated without neutrophils:$\;\mathrm{Viability}\;=\left(\frac{OD550\;sample}{OD550\;control}\right)\times 100.$

### 4.11. Neutrophil Viability Assay

Cell death of neutrophil granulocytes following 60-min co-incubation was measured by the LDH Cytotoxicity Detection Kit (Takara Bio, Shiga, Japan) according to the manufacturer’s instructions. For the measurement of the lactate dehydrogenase (LDH) released into the medium due to the cell death, the cell free supernatant of the cultures was used in a 10-fold dilution. The cell culture supernatant incubated without the hyphae were used as the low control. For high control, neutrophils incubated without the hyphae were lysed with Triton X-100 in a final concentration of 1%. From the data of the low and high controls, a calibration curve was presented, and viability was calculated from the equation of the line.

### 4.12. Statistical Analysis

All measurements were performed in three biological and two technical parallels. Significance was calculated with multiple t-test with False Discovery Rate (FDR) (Q = 10%) in the GraphPad Prism 7 software (GraphPad Software, San Diego, CA, USA).

### 4.13. Ethics Statement

Conforming to the principles outlined in the Declaration of Helsinki, and the healthy volunteers informed consent, blood was taken in the Department of Obstetrics and Gynecology at the University of Szeged, Hungary. The Ethics Committee of the Department of Obstetrics and Gynecology approved the study protocol (20/2016-SZTE).

## 5. Conclusions

Our results show that neutrophils are able to recognize the serum opsonized hyphae of *C. lunata* and can attach to them. They are also activated by a soluble factor produced by the fungus, although induction of oxidative burst is dependent on the presence of the opsonized hyphae. These results suggest the regulatory role of MPO in the immune response against *C. lunata*. Nevertheless, H_2_O_2_ is generated after the activation of the cells. However, oxidative burst seems to be blocked by the fungus after 60 min of interaction. This blockage also influences the NET formation of neutrophils, with which *C. lunata* can efficiently survive the neutrophil attack.
